# A Computational Model of Hopelessness and Active-Escape Bias in Suicidality

**DOI:** 10.5334/cpsy.80

**Published:** 2022-03-31

**Authors:** Povilas Karvelis, Andreea O. Diaconescu

**Affiliations:** 1 Krembil Centre for Neuroinformatics, Centre for Addiction and Mental Health (CAMH), Toronto, Ontario, Canada; 2 University of Toronto, Department of Psychiatry, Toronto, Ontario, Canada; 3 Institute of Medical Sciences, University of Toronto, Toronto, ON, Canada; 4 Department of Psychology, University of Toronto, Toronto, ON, Canada

**Keywords:** suicidality, hopelessness, Pavlovian bias, active-escape bias, active inference, computational modelling, simulation study

## Abstract

Currently, psychiatric practice lacks reliable predictive tools and a sufficiently detailed mechanistic understanding of suicidal thoughts and behaviors (STB) to provide timely and personalized interventions. Developing computational models of STB that integrate across behavioral, cognitive and neural levels of analysis could help better understand STB vulnerabilities and guide personalized interventions. To that end, we present a computational model based on the active inference framework. With this model, we show that several STB risk markers – hopelessness, Pavlovian bias and active-escape bias – are interrelated via the drive to maximize one’s model evidence. We propose four ways in which these effects can arise: (1) increased learning from aversive outcomes, (2) reduced belief decay in response to unexpected outcomes, (3) increased stress sensitivity and (4) reduced sense of stressor controllability. These proposals stem from considering the neurocircuits implicated in STB: how the locus coeruleus – norepinephrine (LC-NE) system together with the amygdala (Amy), the dorsal prefrontal cortex (dPFC) and the anterior cingulate cortex (ACC) mediate learning in response to acute stress and volatility as well as how the dorsal raphe nucleus – serotonin (DRN-5-HT) system together with the ventromedial prefrontal cortex (vmPFC) mediate stress reactivity based on perceived stressor controllability. We validate the model by simulating performance in an Avoid/Escape Go/No-Go task replicating recent behavioral findings. This serves as a proof of concept and provides a computational hypothesis space that can be tested empirically and be used to distinguish planful versus impulsive STB subtypes. We discuss the relevance of the proposed model for treatment response prediction, including pharmacotherapy and psychotherapy, as well as sex differences as it relates to stress reactivity and suicide risk.

## 1 Introduction

Suicide is the second leading cause of death among young adults and among the top ten causes of death across all ages worldwide (Naghavi et al., 2017). Despite decades of research seeking to identify risk factors of suicidal thoughts and behaviors (STB), their predictive ability remains limited (Large et al., 2016; Franklin et al., 2017). Some of the main risk factors include the following: prior psychiatric diagnosis, treatment history, family history of psychopathology, prior self-injurious thoughts and behaviors, substance use and psychosocial stress. However, multivariate suicide risk models based on these factors do not have sufficient sensitivity and specificity in predicting suicide and, even more importantly, lack mechanistic insight to offer clinically useful guidance on selecting optimal individualized interventions (Kessler et al., 2020). As a result, current clinical practice is in need of objective and reliable measures of suicide risk to not have to rely on self-reports, with ∼50% of adults not disclosing their suicidal thoughts and remaining invisible for suicide prevention efforts (Mérelle et al., 2018).

In recent years, cognitive theories have proposed several explanations for the progression from emotional distress to suicidal ideation, and to suicide attempts (Van Orden et al., 2010; Klonsky and May, 2015; O’Connor and Kirtley, 2018; Bryan et al., 2020). At the core of these proposals is the recognition that suicide can be viewed as a means to escape mental pain (psychache) (Baumeister, 1990; Verrocchio et al., 2016). While mental pain and hopelessness contribute to suicidal ideation, other factors, collectively termed ‘acquired capability for suicide’ (e.g., increased physical pain tolerance, access to lethal means), mediate the transition from ideation to suicide attempt (for a review see Klonsky et al. (2018)). While providing useful high-level insights into the different psychological and environmental factors associated with suicidality, the verbal nature of these theories limits their predictive power (Millner et al., 2020; Meehl, 1990). Natural language is inherently vague resulting in intercorrelated constructs on which the theories rest, making it difficult to corroborate or refute them (Millner et al., 2020; Meehl, 1990). This calls for formal theories of suicidality which can be expressed computationally and which can define these constructs operationally (Millner et al., 2020; Dombrovski and Hallquist, 2021). Computational models could allow for a quantification of suicide risk and offer a more mechanistic insight for developing personalized clinical interventions (Nair et al., 2020; Millner et al., 2020). Just as importantly, computational models can help bridge different levels of analysis and establish mechanistic links between behavioral, cognitive, neural and even genetic variables, offering a more integrated understanding of the factors underlying vulnerability to STB (Huys et al., 2021).

One principled way of building such models is to investigate vulnerability to STB through the lens of normative theories of learning and decision making in computational neuroscience (Dombrovski and Hallquist, 2021, 2017). Collectively, STB has been associated with deficits in cognitive control (Richard-Devantoy et al., 2014) and impaired probabilistic learning in the context of rewards and punishments, including impaired delay discounting (Bridge et al., 2015), impaired reversal learning (Dombrovski et al., 2010) and impaired value comparison during the choice process (Dombrovski et al., 2019); for recent reviews see Lalovic et al. (2022) and Sastre-Buades et al. (2021). Outside of the laboratory, this is corroborated by findings of heightened suicide risk in gambling disorders (Karlsson and Håkansson, 2018; Jolly et al., 2021). Behavioral insensitivity to adverse consequences and heightened sensitivity to internal emotional states have also been linked to suicide attempts (Szanto et al., 2014). Together, these findings have led to a proposal of increased Pavlovian over instrumental control as being an important contributing factor to vulnerability to STB (Dombrovski and Hallquist, 2017, 2021). The Pavlovian controller rigidly specifies stimulus-response mappings regardless of outcomes, such as actively escaping proximal threats and avoiding distal threats, resulting in a rather reflexive behavior. In contrast, the instrumental control specifies stimulus-action-outcome mappings enabling one to adapt behaviors to environmental contingencies and maximize desired outcomes, which can be thought of as goal-directed behavior. In line with the idea of increased Pavlovian biases, a recent study by Millner et al. (2019), found STB to be associated with an increased active-escape bias in an Avoid/Escape Go/No-Go task with aversive sound stimuli. In this study, the STB group was more biased towards choosing an active (Go) response in the presence of an aversive sound (in Escape condition), even when withholding the response (in No-Go condition) was the correct response.

**Figure 1:**
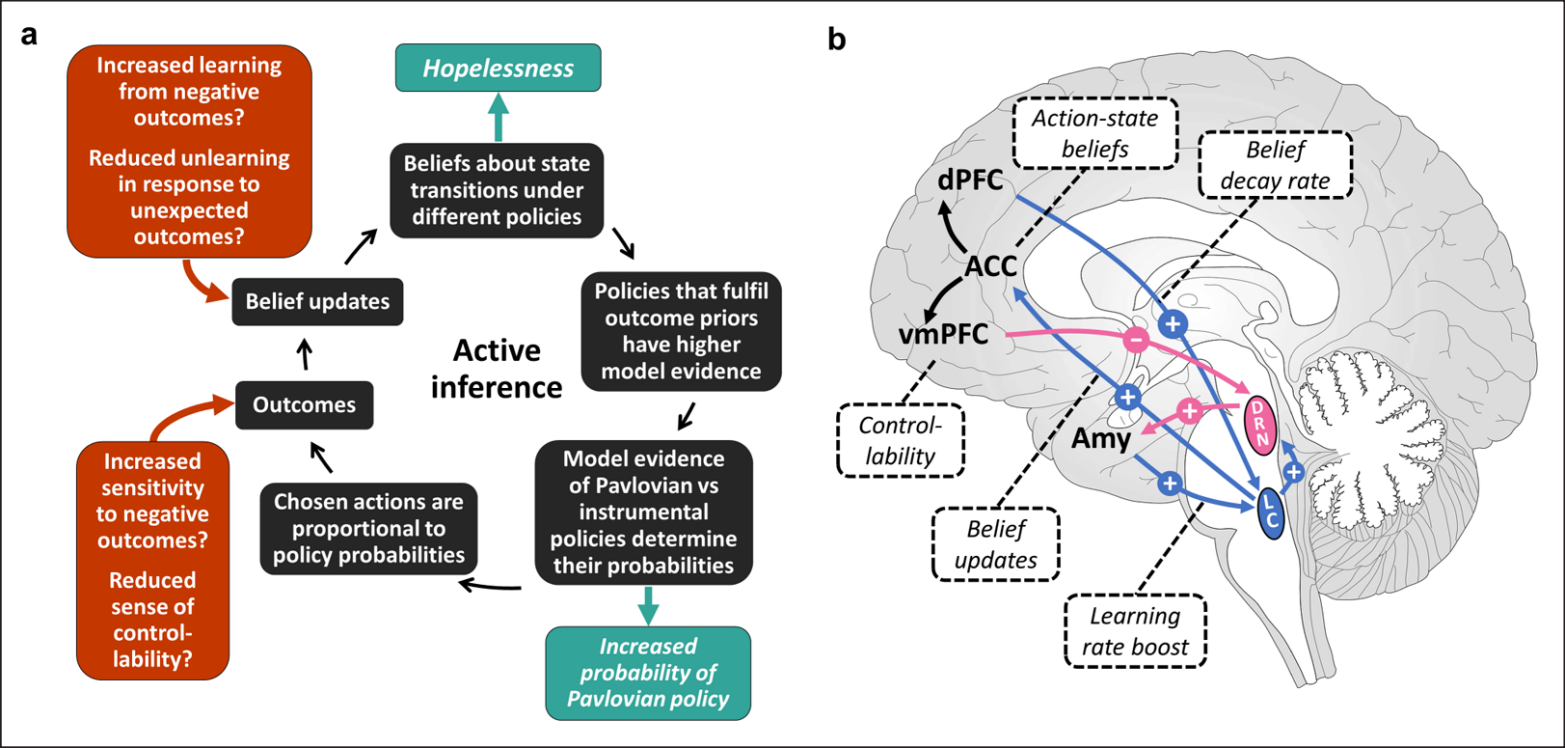
**Hypotheses. (a)** A computational cycle of active inference (black) and potential perturbations at different stages in the cycle (red). These perturbations can give rise to hopelessness – a belief that any taken action will lead to undesired states – and an increased influence of Pavlovian relative to instrumental modes of behavior (teal), both of which are associated with suicidality. (**b**) The brain network that we hypothesize to support the proposed perturbations: norepinephrine modulates belief updates (blue) while serotonin is involved in mediating the effects of stressor controllability (pink). Acute stress leads to increases in the learning rate, which is associated with Amy-LC connectivity (Uematsu et al., 2017; Jacobs et al., 2020), whereas environmental volatility – here assuming state-action prediction errors (SAPEs) as a proxy for environmental change – drives decay of previously learned associations and is mediated by dPFC-LC connectivity (Sales et al., 2019; Clewett et al., 2014). LC projections to the ACC mediate action-dependent state transition belief updates (Tervo et al., 2014; Sales et al., 2019), which are encoded in the ACC (Akam et al., 2021; Holroyd and Yeung, 2012). Finally, controllability of aversive outcomes, which depends on the inferred probabilities of achieving the desired outcomes, reduces aversiveness by inhibiting amygdala activation via the vmPFC-DRN-Amy circuit (Maier and Seligman, 2016; Kerr et al., 2012).

Here, we aim to extend these ideas by proposing a computational mechanism for how the increased Pavlovian biases in STB could result from impaired probabilistic learning (***Figure 1a***). Importantly, we show how this is mediated by hopelessness (a belief that there is nothing one can do to make things better), which is one of the most robust factors of suicide risk (Isometsä, 2014; May et al., 2020). To this end, we apply active inference as the most general neurocomputationally-principled framework that integrates perception, action and learning into a continuous loop of information processing (Friston et al., 2013). The principle guiding this information processing is maximization of (Bayesian) model evidence for one’s model of the world, which simultaneously reduces uncertainty about the world and achieves desired outcomes. We show that by operationalizing hopelessness as predominantly negative instrumental beliefs (i.e., with all available actions believed to have low probability of leading to the desired states), an increased Pavlovian control emerges as a straightforward consequence of the drive to maximize model evidence. We propose four different perturbations that within the context of aversive learning could give rise to hopelessness itself: (1) an increased learning from aversive outcomes, (2) a reduced belief decay in response to unexpected outcomes, (3) an increased stress sensitivity and (4) a reduced sense of stressor controllability.

Importantly, these proposals stem from the consideration of neurocircuits implicated in STB. Research on suicide neuromarkers point to the circuits underlying stress response, implicating the locus coeruleus – norepinephrine (LC-NE) and the dorsal raphe nucleus – serotonin (DRN-5-HT) systems (Mann and Rizk, 2020; Oquendo et al., 2014; van Heeringen and Mann, 2014). More broadly, neuroimaging findings are converging on fronto-limbic regions involved in emotion regulation and cognitive control, including the amygdala (Amy), the anterior cingulate cortex (ACC), the dorsal prefrontal cortex (dPFC) and the ventromedial prefrontal cortex (vmPFC) among other regions (Schmaal et al., 2020; Balcioglu and Kose, 2018). However, computational models linking these neuromarkers with the behavioral markers are still missing. Here we suggest that our proposed computational perturbations in STB could be related to how the LC-NE together with the Amy, the dPFC and the ACC mediate learning in response to acute stress and volatility as well as how the DRN-5-HT together with the vmPFC regulate stress responses based on the perceived controllability of the aversive stimulus (***Figure 1b***).

To validate our model, we run model simulations in a probabilistic Avoid/Escape Go/No-Go task, demonstrating how the proposed perturbations lead to hopelessness, increased Pavlovian control and increased active-escape bias – replicating recent empirical findings by Millner et al. (2019). This serves as a proof of concept and produces a computational hypothesis space which can be investigated experimentally and which might speak to different subtypes of suicidal behaviour: impulsive versus planful attempts (Schmaal et al., 2020; Dombrovski and Hallquist, 2017; Bernanke et al., 2017).

## 2 Materials and Methods

### 2.1 Modelling rationale

#### 2.1.1 Behavioral control

Within the active inference framework, Pavlovian and instrumental modes of behavior can be derived from the same central computational goal, which could be thought of as maximizing model evidence, resisting entropy or maintaining homeostasis (Pezzulo et al., 2015). Being nested hierarchically – from reflexive to Pavlovian, to habitual, to instrumental behaviors – different modes of behavior allow for the successful navigation of increasingly more complex environments, but also require more computational and metabolic resources. This poses a problem of bounded rationality (i.e. finding a balance between behavioral accuracy and metabolic costs), which can be resolved by performing Bayesian model averaging (BMA) over the different modes of behavior (FitzGerald et al., 2014). This means that actions are informed by all modes of behavior, whereby the modes with the highest model evidence have the most influence. In these computational terms, a stronger active-escape bias in suicidality can be understood as resulting from a reduced model evidence for instrumental relative to Pavlovian control.

In active inference, the model evidence of different policies (e.g., Pavlovian vs. instrumental) depends on how well they are expected to result in desired outcomes (Friston et al., 2016). Thus, saying that instrumental control has a reduced model evidence is the same as saying that instrumental control is expected to have a reduced probability of fulfilling desired outcomes – i.e., beliefs are more ‘negative’, not mathematically (not below zero), but colloquially speaking. Here we operationalize hopelessness, which is one of the most robust suicide risk factors (May et al., 2020; Isometsä, 2014), as strong negative instrumental beliefs about state transitions.

#### 2.1.2 Learning: uncertainty, stress and norepinephrine

To understand how hopelessness arises, we have to consider the dynamics of belief updating, i.e. learning. Having predominantly negative beliefs (hopelessness) implies either a predominantly aversive environment or preferential learning from aversive events. Asymmetries in how positive and negative outcomes drive learning (i.e. affective bias) have been implicated in mood disorders (Pulcu and Browning, 2017; Clark et al., 2018; Pulcu and Browning, 2019), with negative outcomes having larger effect on learning than positive outcomes (Mathews and MacLeod, 2005; Eshel and Roiser, 2010). Conversely, in the general population learning is driven more strongly by positive outcomes (Sharot and Garrett, 2016). In STB, research on learning from negative vs. positive outcomes is scarce, but a recent study showed STB to be associated with faster processing of negative stimuli (Harfmann et al., 2019).

While the learning rate can be affected by multiple neuromodulatory systems, when it comes to adjusting the learning rate in response to acute stress and volatility, the LC-NE system plays a central role (Pulcu and Browning, 2019; Cook et al., 2019; Silvetti et al., 2018; Jepma et al., 2016; Lawson et al., 2020). Previous influential theories of LC function were founded on the assumption that LC-NE cells behave homogeneously (Yu and Dayan, 2005; Bouret and Sara, 2005). However, recent research emphasizes that LC firing properties are not topographically homogeneous and rather that the LC is comprised of largely non-overlapping target-specific subpopulations of neurons (Poe et al., 2020; Chandler et al., 2019). Importantly, aversive learning is mediated by Amy-LC connectivity (Sterpenich et al., 2006; Uematsu et al., 2017; Jacobs et al., 2020), whereas connectivity between the prefrontal cortex (PFC) regions and the LC has been found to represent ‘unlearning’, which is necessary for faster adaptation to environmental change or volatility (Uematsu et al., 2017; Sales et al., 2019). Relevant for our aims here, dPFC-LC connectivity has been shown to encode learning from unpredictable feedback (Clewett et al., 2014) and response conflict resolution (Köhler et al., 2016; Grueschow et al., 2020). The dorsolateral PFC (dlPFC) itself has been associated with state prediction error (as opposed to reward prediction error) (Gläscher et al., 2010). LC projections to the ACC have been shown to mediate updates of action-dependent beliefs about the environment (Tervo et al., 2014; Sales et al., 2019), with the ACC encoding such beliefs (Akam et al., 2021; Holroyd and Yeung, 2012). This is consistent with the findings that ACC activity correlates with reward expectation, prediction errors, learning rate and volatility (Rushworth and Behrens, 2008), with these learning variables engaging the ACC primarily in the context of learning about the value of instrumental actions (Matsumoto et al., 2007).

Several lines of evidence suggest the aforementioned networks to be implicated in suicidality (Schmaal et al., 2020; Oquendo et al., 2014). Studies have reported fewer LC neurons, LC overactivity and depletion of NE, all of which are thought to be associated with a dysregulated stress response (Oquendo et al., 2014; van Heeringen and Mann, 2014). The Amy is reported to show increased resting state functional connectivity (Kang et al., 2017) with some structural MRI studies also reporting larger Amy volumes (Monkul et al., 2007; Spoletini et al., 2011). Studies on the dPFC report reduced volumes (Ding et al., 2015), decreased resting regional cerebral blood flow (rCBF) (Willeumier et al., 2011) and reduced activation during error processing (Vanyukov et al., 2016). ACC volumes are also reported to be reduced, with reductions in rostral ACC (rACC) being most significant (Wagner et al., 2011). In a risk aversion task, suicide attempters showed a blunted subgenual ACC activation in response to potential gains (Baek et al., 2017), a reduced ACC response to sad faces and an increased response to wins versus loses (Olié et al., 2015). Finally, a recent study found greater rACC-Amy functional connectivity to be associated with suicidal ideation and previous suicide attempts (Alarcón et al., 2019).

Here, we propose that a disruption in any part of the Amy-dPFC-LC-ACC network (***Figure 1b***, blue) could lead to hopelessness, increased Pavlovian and active-escape bias, increasing the risk of STB. Specifically, we consider two possible perturbations. First, an increased Amy response to negative outcomes would increase learning from negative outcomes (i.e., negative affective bias), which may lead to more negative beliefs (hopelessness) and thus stronger Pavlovian influences. This is supported by increased learning rate in STB observed in an aversive learning task (Millner et al., 2019). Second, reduced activity in the dPFC in response to state-action prediction errors would result in less belief decay allowing negative experiences to accumulate, thus also resulting in hopelessness and stronger Pavlovian biases. Interestingly, impairments in the dPFC have been mostly associated with planful suicides (Schmaal et al., 2020), which would be in agreement with the cognitive rigidity induced by reduced belief decay that we consider here.

#### 2.1.3 Controllability: stress and serotonin

Recent work has shown that controllability of action outcomes governs arbitration between Pavlovian and instrumental control in line with BMA (Dorfman and Gershman, 2019). These effects were found to be associated with frontal midline theta power, which suggests involvement of the mPFC and the ACC (Csifcsák et al., 2020). Furthermore, it has been proposed that dorsal ACC (dACC) could be understood as encoding the expected value of control (Shenhav et al., 2013). This is very similar to what we have proposed in relation to hopelessness. Indeed, *controllability* and *hopelessness* are very closely related constructs. Uncontrollable aversive stimulation has been used to study learned helplessness, from which the construct of hopelessness has been derived (Liu et al., 2015). Another extensively studied effect of controllability is that of modulating the stress response. Stressor controllability has been associated with the vmPFC-DRN-Amy network, and thus with 5-HT-modulated stress response (Maier and Seligman, 2016; Kerr et al., 2012; Hiser and Koenigs, 2018). More specifically, stressor controllability activates the vmPFC, which then inhibits DRN, which in turn reduces amygdala activation in response to a stressor (Maier and Seligman, 2016). Relevant for our aims here, recent studies also show this effect to be associated with successful instrumental learning (Collins et al., 2014; Wanke and Schwabe, 2020).

Considering these findings, we introduce a computational distinction between hopelessness and controllability. As we have defined it earlier, hopelessness corresponds to negative instrumental state-action beliefs that are encoded in the ACC and are arrived at via LC-mediated updates. Controllability, on the other hand, we associate with the vmPFC-DRN-Amy network and thus with 5-HT-modulated stress response. Instrumental state-transition beliefs encoded in the ACC underlie inference about future states and future outcomes, which are then used to estimate controllability in the vmPFC (see Model implementation section for more detailed rationale). This provides a computational link between the NE-modulated and the 5-HT-modulated variables and allows hopelessness and controllability to be distinct but coupled. Interestingly, projections from the LC to the DRN have also been shown to regulate 5-HT release (Pudovkina et al., 2003) and be necessary for developing learned helplessness following uncontrollable stressor exposure (Grahn et al., 2002), providing another point of interaction between the two neuromodulatory systems, which we do not specifically address here.

In suicidality, a large body of research points to deficits in the serotonergic system (van Heeringen and Mann, 2014; Oquendo et al., 2014). While lower 5-hydroxyindoleacetic acid (5-HIAA) levels – a major serotonin metabolite – in the cerebrospinal fluid (CSF) suggest reduced overall serotonergic activity (Mann et al., 2006), serotonin in the brainstem is found to be elevated (Bach et al., 2014), with serotonergic action being elevated in the DRN due to less reuptake (Arango et al., 2001). Furthermore, studies also report elevated serotonin binding in the Amy (Hrdina et al., 1993) and fewer serotonin transporters in the vmPFC and the ACC (Mann et al., 2000). A recent study has also found a history of suicide attempts to be associated with a diminished functional connectivity between vmPFC and Amy (Wang et al., 2020). Together, these findings are consistent with an increased 5-HT-mediated stress response in suicidality.

Here we propose that a reduced sense of controllability stemming from vmPFC-DRN-Amy network impairments (***Figure 1b***, pink) can lead to a stronger Amy activation in response to stress, thus increasing learning from negative outcomes and leading to hopelessness and stronger Pavlovian biases. Impairments in the vmPFC have been associated with impulsive suicide attempts (Schmaal et al., 2020), which would be in line with larger belief updates in response to stressors.

### 2.2 Model implementation

**Figure 2:**
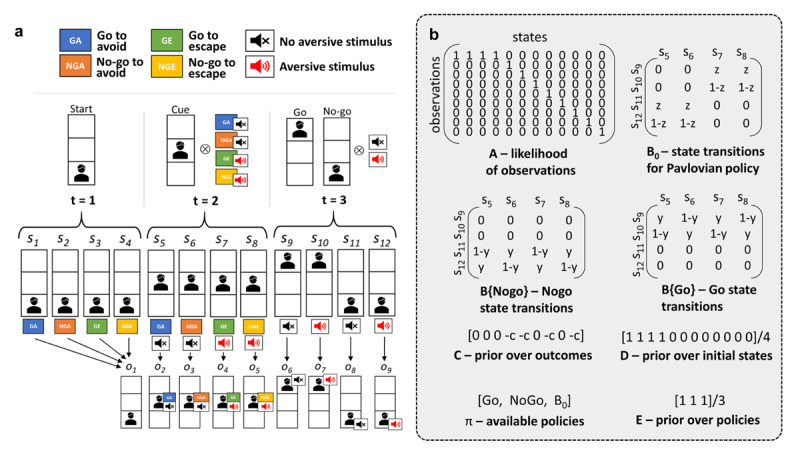
**Avoid/Escape Go/No-Go task design and model specification.** (**a**) Following Millner et al. (2018; 2019) the task has 4 cues corresponding to the 2 × 2 (Go/No-Go x Avoid/Escape) factorial task structure, with 2 possible outcomes: aversive or neutral. For modelling purposes, the task was divided into 3 discrete time points. At the start of a trial (*t =* 1) the agent is in one of the four hidden states (*s*_1–4_) and no observations are available (*o*_1_). Next, the agent is taken to *t =* 2, where a cue (and in the case of Escape condition also an aversive sound) is presented corresponding to one of four possible hidden states (*s*_5–8_) and observations (*o*_2–5_). At *t =* 2 the agent chooses what action to take (Go or No-Go) which then leads to one of four possible states (*s*_9–12_) and observations (*o*_6–9_): Go response + silence (*s*_9_, *o*_6_), Go response + aversive sound (*s*_10_, *o*_7_), No-Go response + silence (*s*_11_, *o*_8_), No-Go response + aversive sound (*s*_12_, *o*_9_). (**b**) The main model structures. The likelihood of observations, **A**, was implemented to have deterministic mappings between states and observations due to the salience of the aversive stimulus and the cues. As no learning was required, **A** in the generative model and in the generative process were identical. State transitions from *t =* 2 to *t =* 3 for instrumental (Go/No-Go) policies B were probabilistic, captured by the *y* parameter. For the objective transition probabilities *y* was set to 0.8, meaning that correct response by the agent led to the neutral state 80% of the time. For the generative model, *y* was initialized with 0.5 to correspond to the agent having a uniform prior over the two possible transitions. The zero probabilities for the other transitions reflect the assumption that the agent understands the task structure and does not expect to end up in a Go state after choosing No-Go and vice versa. State transition probabilities from *t =* 2 to *t =* 3 for the Pavlovian policy, **B**_0_, were implemented to allow only for No-Go responses in the Avoid and Go responses in the Escape conditions (with Go responses in the Avoid and No-Go responses in the Escape conditions having 0 probabilities). The strength of the belief that the Pavlovian policy will lead to the desired states is captured by the *z* parameter. Prior over outcomes (**C**) assumed that the agent does not like outcomes 4, 5, 7 and 9 (all of which involve the aversive stimulus). The strength of this preference of neutral outcomes is captured by parameter *c*. The prior over initial states **D** was assumed to be uniform for states 1–4. The other states have zero probability, which reflects the assumption that the agent understands the task structure and does not expect to be in states 5–12 at the beginning of a trial. Finally, prior over policies **E** was also assumed to be uniform across the available Go, No-Go and Pavlovian policies (*π*). See **S1 Appendix: full mathematical details of the model** for more implementation details.

In the previous sections we have laid out a conceptual picture of our proposed model by considering various computational and neurobiological findings. In this section, we will present one possible computational implementation by focusing on an Avoid/Escape Go/No-Go task (***Figure 2a***). Note that the implementation of the model is not at the level of neural dynamics but rather at the higher level of computational mechanisms underwritten by such dynamics (cf. Marr’s levels of analysis (Marr and Poggio, 1976)). However, the active inference framework has deep connections to neurobiology and has recently been applied to understanding a whole range of psychiatric conditions (Smith et al., 2021), including the effects of noradrenergic and serotonergic drugs in depression (Constant et al., 2021).

The task employs a 2 × 2 (Go/No-go x Avoid/Escape) factorial design. On every trial, the agent is presented with one of four cues. Two of the cues are always paired with an aversive sound (Escape condition) while the other two are paired with silence (Avoid condition). The agent’s goal is to learn, for each cue, which response (active Go or passive No-go) more frequently results in silence during feedback. For ease of reference, we will refer to the responses that maximize the frequency of silence during the feedback as the “correct” responses throughout the paper. This means that in the Avoid condition, correct responses will prevent the aversive sound from playing, while in the the Escape condition, correct responses will stop the aversive sound that is already being played. However, the feedback is probabilistic, which means that even “correct” responses will sometimes lead to experiencing the aversive sound. Probabilistic feedback introduces uncertainty and makes it more challenging to learn, which response is correct.

To model this task, we use an active inference scheme for discrete Markovian models (Friston et al., 2016), which means that we will be dealing with *discrete* time steps (*t*), states (*s*), actions (*a*), and observations (*o*). Each trial gets divided into three time steps. At *t =* 1, the agent is in one of four possible hidden states with no informative observations about the task conditions available (e.g., a fixation cross is displayed, which does not contain any information on which cue will be presented next). At *t =* 2, the agent is presented with one of the four cues, which correspond to one of the four conditions resulting from the 2 × 2 (Go/No-Go × Avoid/Escape) factorial design. In the Avoid condition, there is no aversive sound while the agent is choosing either a Go or No-Go response. In the Escape condition, the aversive sound is present throughout the decision phase. At *t =* 3, the agent observes the final outcome of a trial, either aversive or neutral. This means that in the Avoid condition, a correct action leads to no aversive sound, while in the Escape condition, a correct action results in the discontinuation of the aversive sound. When choosing an action at *t =* 2, the agent relies on available policies *π*: instrumental Go/No-Go and Pavlovian. Probabilities of these policies depend on the underlying beliefs about likelihood of observations, **A**, state transitions – **B**{Go}, **B**{No-Go}, **B**_0_ – as well as prior beliefs over outcomes (i.e., preferences), **C**. In other words, probabilities of policies depend on model evidence that each set of beliefs provides, where model evidence is approximated with variational free energy (see **S1 Appendix: full mathematical details of the model** and Da Costa et al. (2020) for more details).

After each trial, the agent updates their beliefs depending on the outcome in that trial. Since there is no ambiguity about observations due to their saliency, we assume all learning to concern only state transition probabilities (**B**). Columns in **B** matrices are Dirichlet distributions parameterized with concentration parameters ***b***, such that for a control state *u*, ***B***(*u*) = *Dir*(***b***(*u*)). Concentration parameters can be interpreted as the number of times various combinations of state transitions have been observed, which effectively captures both the probability and the confidence in that probability. At the end of each trial, state transition concentration parameters are updated via:


1 \[
{b_i}(u) = {b_{i - 1}}(u) + \eta \sum\limits_{\tau ,p} {\pi _{\tau -1}^p} \;s_\tau ^p \otimes s_{\tau -1}^p - \frac{{{b_{i - 1}}(u) - 1}}{\lambda }\;,
\]

where *i* denotes the trial number, *u* denotes the control state (Go or No-Go) and \[
s_\tau ^p
\] contains posterior probabilities of different states under each policy *p* for time point *τ*. Note that in the current implementation, we only care about *τ* = 3, because the transition between *t =* 1 and *t =* 2 does not depend on the agent’s choices. *π* denotes posterior policy probabilities.

The sum in the second term of the equation is performed only over the two instrumental policies. This means that in an extreme case where behavior is driven primarily by the Pavlovian policy and the probabilities of instrumental policies are very low, there will be very little learning even though the agent has the information to update their beliefs about state transitions. To account for instrumental learning facilitated by Pavlovian responses (Holmes et al., 2010), one could consider combining the posterior probabilities of Pavlovian Go or No-Go responses with instrumental Go and No-Go policy probabilities, respectively, when updating beliefs about controlled state transitions. However, in the simulations presented in this paper, Pavlovian effects are never too extreme and similar results can be obtained with either implementation. To keep the model simpler, here we present the results using the original implementation where the sum in the second term of the equation is performed only over the two instrumental policies.

The remaining two parameters *η* and *λ* in **Eq. (1)** control the learning rate and the decay rate, respectively. The learning rate controls how much new experiences add to the existing concentration parameters, while the decay rate controls how much the previously accumulated concentration parameters should be discounted. Without the decay factor, concentration parameters would accumulate indefinitely making the agent too rigid and thus too slow to adapt if environmental contingencies were to change. Following the work of Sales et al. (2019), *λ* is assumed to depend on state-action prediction errors (SAPEs) and to be associated with effective connectivity from the dPFC to the LC. This makes the decay factor sensitive to environmental volatility: changing environmental contingencies will result in larger SAPEs, which in turn will speed up unlearning of no longer accurate beliefs, allowing the agent to learn the new contingencies faster. The relationship between SAPEs and *λ* is modelled using a logistic function:


2 \[
\lambda = {\lambda _{min}} + \frac{{{\lambda _{max}} - {\lambda _{min}}}}{{1 + {e^{\;g(SAPE - m)}}}}\;,
\]

where *g* is the gradient, *m* is the midpoint, while *λ_min_* and *λ_max_* are minimum and maximum function values. Note that higher SAPEs will result in a smaller *λ*, which will result in more belief decay because *λ* is a denominator in the update equation **Eq. (1)**. SAPE itself is defined as Kullback-Leibler (KL) divergence between BMA distributions at successive time steps:


3 \[
SAPE(t) = {D_{KL}}[(S_\tau ^t)||S_\tau ^{t-1})]\;\;.
\]

In the simulations presented in this paper, SAPE is computed for t = 3, after the action (Go/No-Go) is performed and only for predictions about the final states (*τ* = 3). BMAs themselves are computed via:


4 \[
{S_\tau } = \sum\limits_p {\pi _\tau ^p} \cdot s_\tau ^p\;,
\]

where \[
\pi _\tau ^p
\] denotes posterior policy probabilities and \[
s_\tau ^p
\] denotes posterior state probabilities for policy *p* at time point *τ*.

In addition to being sensitive to environmental change (i.e. volatility), the LC-NE system also coordinates aversive learning mediated by Amy-LC connectivity (Uematsu et al., 2017; Jacobs et al., 2020). To capture these effects, we introduce a learning rate dependency on outcome valence (assuming Amy activation during aversive outcomes), which we associate with the preference against aversive outcomes encoded in the **C** vector:


5 \[
\eta = 1 + k|C(o)|\;,
\]

where *C(o)* is the value of prior preference for outcome *o*, with the parameterization being *-c* for the aversive stimulus outcomes and *0* for the neutral outcomes. Parameter *k* is a scaling factor that could correspond to effective connectivity between the Amy and the LC. Note that the learning rate dependence on valence that we introduce here is what enables the model to account for affective biases (Pulcu and Browning, 2017; 2019; Sharot and Garrett, 2016; Eshel and Roiser, 2010). A more principled implementation of valence and its role in modulating the learning rate could depend on the rate of change of free energy over time (Joffily and Coricelli, 2013).

The final component of the model aims to account for how controllability of aversive outcomes inhibits Amy activation via the serotonergic system involving vmPFC-DRN-Amy network (Maier and Seligman, 2016; Kerr et al., 2012). We implement this by modulating stress sensitivity parameter *c* by a controllability parameter *w*:


6 \[
c^{\prime} = {c^{(1 - w)}}
\]

In the limiting cases when there is no control (*w =* 0), *c*′ is equal to the original *c* and when there is complete control (*w =* 1) *c*′ is equal to 1. Controllability itself is assumed to depend on on beliefs that the neutral outcome can be reached:


7 \[
{w_n} = \sum\limits_{i = 6,8} {o_{\tau = 3}^{t = 2}} ({o_i})\;,
\]

where \[
o_{\tau = 3}^{t = 2}
\] contains expected outcomes at time point *τ* = 3 at time *t =* 2; and here we are summing over the two possible neutral outcomes (*o*_6_ and *o*_8_) for time point *τ* = 3. Expected outcomes are simply a product of the likelihood of observations **A** and BMA expected states ***S**τ*. This means that the subjective estimate of controllability depends on inferred and expected states, and not on actual states of the world. Parameter *w*_n_ effectively represents an average probability of achieving the desired outcome in the inferred context. Note that this is similar to the well-established finding of vmPFC encoding expected value (see Hiser and Koenigs (2018) for a review). Furthermore, such distinction between vmPFC, which encodes expected outcome (which we associate with controllability), and ACC, which encodes state-transition probabilities (which we relate to hopelessness) is consistent with the finding that vmPFC encodes stimulus-based value and is more active during the outcome phase (cf. stress response) and that ACC encodes action-based value and is more active during both outcome and decision phases (cf. instrumental control and learning) (Vassena et al., 2014). The close relationship between the subjective feeling of control and outcome valuation has also been demonstrated in recent studies (Stolz et al., 2020; Wang and Delgado, 2019). Relevantly, STB has been associated with reduced activation to expected value in vmPFC (Brown et al., 2020; Dombrovski and Hallquist, 2017).

Finally, to collectively account for any impairments of how *w*_n_ modulates the stress response (i.e., any impairments along the vmPFC-DRN-Amy network), we transform *w*_n_ into the final estimate of controllability by entering it into a logistic function constrained by a controllability threshold *w*_0_ (i.e. the midpoint of the logistic function) and a gradient *g*_w_:


8 \[
w = \frac{1}{{1 + {e^{\; - {g_w}({w_n} - {w_0})}}}}\;.
\]

The dependency of the learning rate on stress sensitivity (*c*), means that controllability can indirectly regulate learning rate through its effects on stress sensitivity. This is in line with recent findings showing that DRN serotonin neurons modulate the learning rate and they do so in proportion to uncertainty about decision outcomes (Grossman et al., 2021), which is also in agreement with how we implemented controllability (**Eq. (7)**).

Note that even though we have provided a reasonable theoretical justification for introducing the controllability component, it is a rather ad hoc addition to the otherwise computationally principled active inference framework. It is important to stress, however, that the simulation results that we present in the next section do not hinge on this additional computation, except for the results concerning the controllability parameter itself.

**Figure 3:**
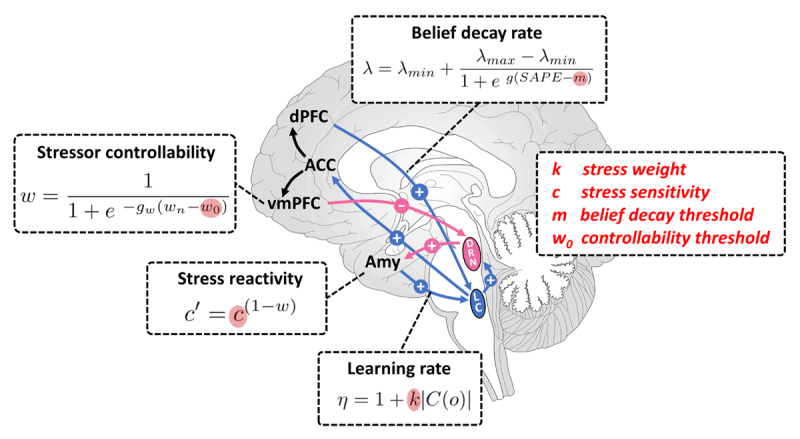
**Summary of the proposed computations, possible neural correlates and parameters of interest for STB.** Within the proposed model there are four areas of relevance for STB: learning rate, belief decay rate, stress reactivity and perceived controllability of a stressor. Stress weight parameter, *k*, controls the boost in the learning rate in response to stress. Increasing this parameter would result in increased learning from stressful outcomes. Stress sensitivity parameter, *c*, captures individual sensitivity to stress, which then also affects the learning rate. Controllability threshold, *w*_0_, is a midpoint in the logistic function that translates the beliefs about state transitions into an estimate of stressor controllability. In other words, *w*_0_ regulates how positive state transition beliefs have to be for a stressor to be deemed sufficiently controllable. Finally, belief decay threshold, *m*, regulates how large state-action prediction errors (SAPEs) have to be before significant belief decay takes place. Note that for the decay rate and the controllability there are other parameters (gradients, *g_w_, g*, and minimum and maximum decay values λ_min_, λ_max_) that we could inspect, but for simplicity here we focus on the midpoint values *w*_0_ and *m* as the exact parameterization of these effects is somewhat arbitrary and the midpoints are sufficient for exploring the general direction of different manipulations.

***Figure 3*** summarizes the proposed computations as well as their possible neural correlates and highlights parameters of interest for STB.

## 3 Results

### 3.1 Model simulations

**Figure 4:**
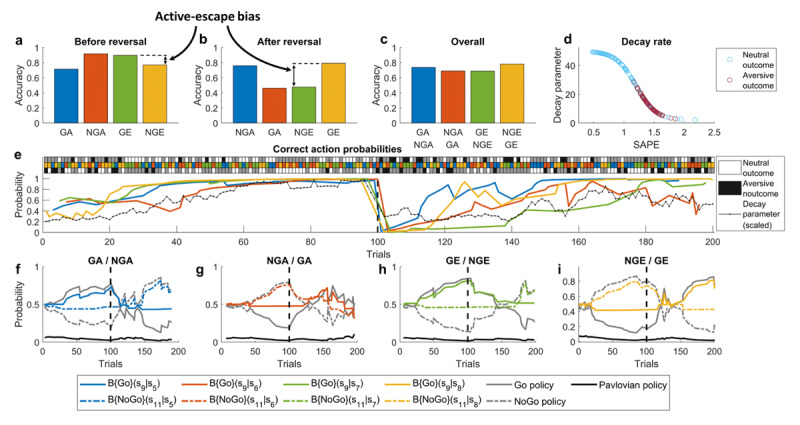
**Model simulations: a single (healthy control) participant with low stress weight (*k* = 0.1). (a-c)** average choice accuracy before reversal, after reversal and overall, respectively, for Go-to-Avoid (GA), No-Go-to-Avoid (NGA), Go-to-Escape (GE) and No-Go-to-Escape (NGE); the four colors denote different cues used in the task. The results in (**a**) reproduce active-escape bias reported by Millner et al. (2018) in the general population. (**b**) and (**c**) are additional predictions about performance after reversal and overall, respectively. (**d**) Decay parameter values for different SAPEs throughout the task. Note that SAPEs for aversive outcomes are larger which leads to smaller decay parameter, and thus to larger belief decay (see **Eq. (1)**). (**e**) Performance across all trials. The top 3-row panel shows the sequence of cue presentation (middle row), executed action (non-grey squares: bottom row – No-Go, top row – Go) and trial outcome (white – neutral, black – aversive); each column corresponds to a single trial. Actions are represented implicitly by either black or white color. If for a given trial the top square is either black or white, it means that the Go action was selected, if the bottom square is either black or white then the No-Go action was selected. The main panel shows trajectories of correct action probabilities, which gradually increase as the task progresses, but drop sharply once the Go/No-Go cue meanings are reversed on trial 100. The response to this environmental change can be seen in the decreased decay parameter (black line), which drives faster forgetting of previously learned contingencies and allows the agent to adapt. Note that decay parameter trajectory here is scaled to be between 0 and 1 and smoothed out using moving average with a window size of 5 trials. (**f-i**) Trajectories of underlying beliefs about state transitions and policy probabilities. These plots reflect the straightforward relationship between belief strength and policy probability: as the probability of an instrumental Go/No-Go action leading to the desired state increases (solid/dash-dotted colored lines) the probability of choosing Go/No-Go policy tracks that increase (solid/dash-dotted gray), and probabilities of Pavlovian policies (solid black) decrease as a result. The vertical dashed lines in all of the plots denote the reversal.

**Figure 5:**
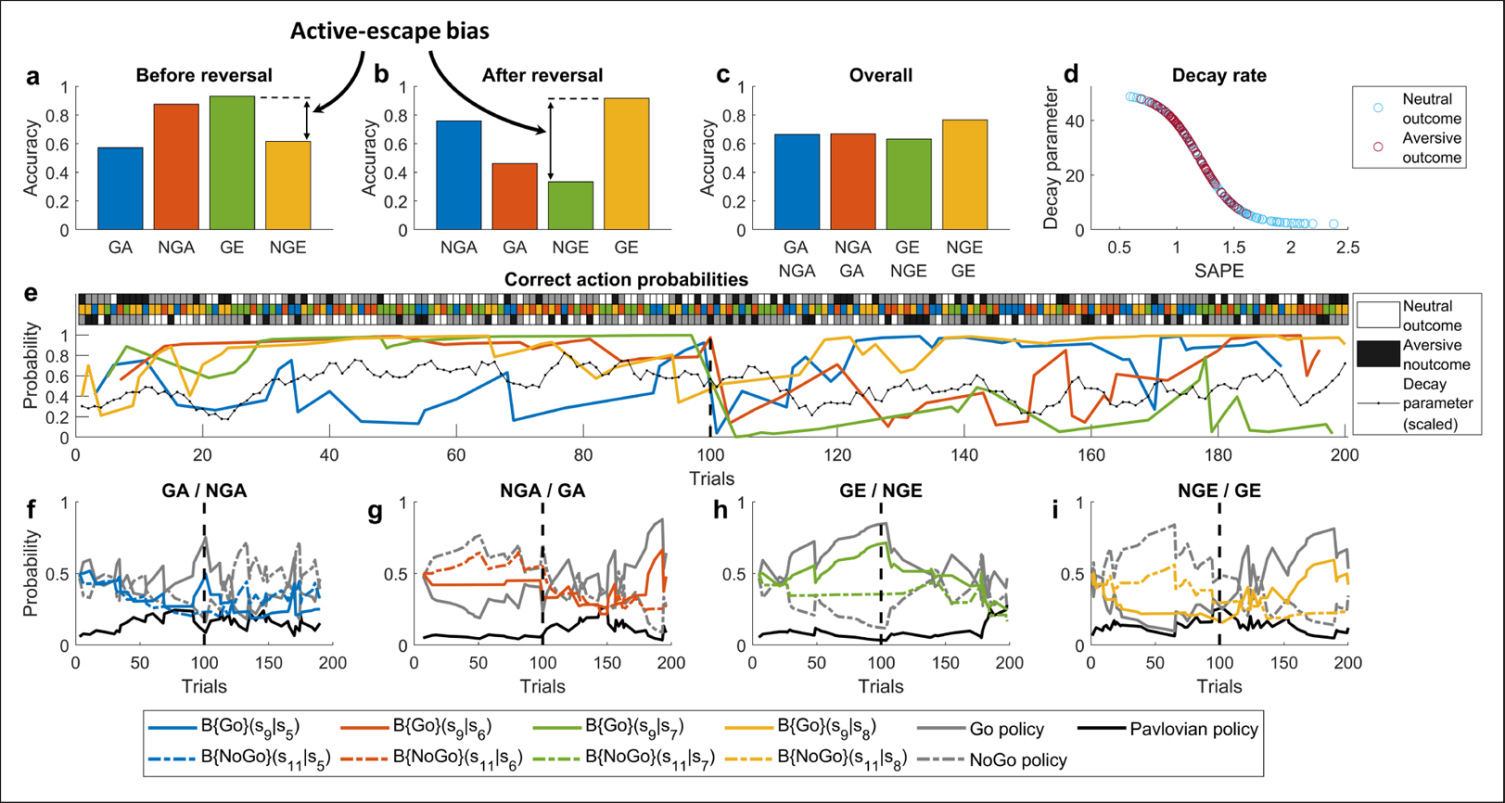
**Model simulations: a single (STB) participant with a high stress weight (*k* = 1). (a-c)** average choice accuracy before reversal, after reversal and overall, respectively, for Go-to-Avoid (GA), No-Go-to-Avoid (NGA), Go-to-Escape (GE) and No-Go-to-Escape (NGE); the four colors denote different cues used in the task. The results in (**a**) reproduce increased active-escape bias in suicidality reported by Millner et al. (2019), and predict that this bias would be even larger after a reversal in cue meanings (**b** panel). (**d**) Decay parameter values for different SAPEs throughout the task. Note that now aversive outcomes produce smaller SAPEs, due to increased expectation of aversive states. (**e**) Performance across all trials. The top 3-row panel shows the sequence of cue presentation (middle row), executed action (non-grey squares: bottom row – No-Go, top row – Go) and trial outcome (white – neutral, black – aversive); each column corresponds to a single trial. Actions are represented implicitly by either black or white color. If for a given trial the top square is either black or white, it means that the Go action was selected, if the bottom square is either black or white then the No-Go action was selected. The main panel shows trajectories of correct action probabilities. Compared to the healthy control in the previous figure, the trajectories are noisier, especially after the reversal on trial 100. Decay rate trajectory (black line) is also nosier, which is partly responsible for the poor adaptation after the reversal. Note that decay parameter trajectory here is scaled to be between 0 and 1 and smoothed out using moving average with a window size of 5 trials. (**f-i**) Trajectories of underlying beliefs about state transitions and policy probabilities. Compared to the healthy control, the belief trajectories are noisier, but even more importantly, beliefs about the instrumental transitions to neutral states are on average weaker (cf. hopelessness), which leads to increased probability of the Pavlovian policy. The vertical dashed lines in all of the plots denote the reversal.

**Figure 6:**
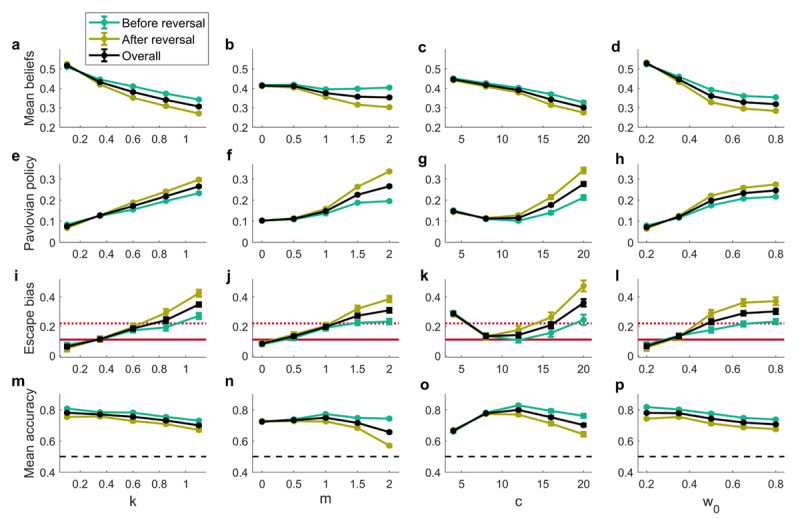
**Model simulations: exploration of the hypothesis space.** Each column shows the effects of varying on of the parameters: *k* – stress weight (while *m =* 1.3, *c =* 8, *w*_0_ = 0.6), *m* – belief decay threshold (while *k =* 0.7, *c =* 8, *w*_0_ = 0.6), *c* – stress sensitivity (while *k =* 0.6 *m =* 1, *w*_0_ = 0.5) and *w*_0_ – controllability threshold (while *k =* 0.9, *m =* 1.3, *c =* 8). (a-d) the mean of beliefs that the neutral state will be reached averaged across 4 contexts and 2 possible actions. (**e-h**) The mean probability of choosing the Pavlovian policy. (**i-l**) Active-escape bias (the difference between choice accuracy on GE and NGE trials). The solid and dashed red lines denote the expected active-escape bias in healthy control group and suicidality group, respectively (based on Millner et al. (2018; 2019) findings). (**m-p**) Mean choice accuracy across all 4 contexts.

To validate the model, we first simulated performance on the task for a single healthy control (***Figure 4***), and then showed how increasing parameter *k* – which regulates aversiveness-related component of the learning rate and is assumably represented in terms of Amy-LC connectivity – can produce increased active-escape biases and other behavioral and cognitive aspects associated with suicidality (***Figure 5***). Finally, we defined a wider hypothesis space, exploring how different parameters in the model can independently lead to the behavior observed in STB. (***Figure 6***).

For the initial simulations (***Figures 4 and 5***), we ran 200 trials of the task, where at every trial one of the 4 cues was presented at random. After 100 trials, the meanings of the cues were reversed: Go becoming No-Go and vice versa. In this simulation, the model parameters were set to *k =* 0.1, *m =* 1.3, *c =* 8, *w*_0_ = 0.5, *z =* 0.4, *λ_min_* = 2, *λ_max_* = 50, *α* = 3 and *β* = 1 to produce reasonable performance trajectories as well as an active-escape bias (***Figure 4a***) consistent with empirical findings reported by Millner et al. (2018). As the agent’s beliefs approach the actual state transition probabilities (***Figure 4f-i***, colored lines), this makes the neutral outcomes more expected, thus invoking only small SAPEs in contrast to unexpected aversive outcomes (***Figure 4d***). This is also what drives successful unlearning after the reversal: a series of negative outcomes with large SAPEs result in a sharp drop in the decay parameter (***Figure 4e***, black line), which increases belief decay and facilitates quick learning of new contingencies. The Pavlovian policy that underlies the active-escape bias can be seen at its strongest at the very beginning of the task and right after the reversal, when beliefs that instrumental actions will lead to neutral outcomes are lower (***Figure 4f-i***).

By increasing parameter *k* to 1, the size of the belief update after experiencing aversive outcomes becomes larger, reproducing the increased active-escape bias (***Figure 5a***) by a similar magnitude as reported in individuals with STB (Millner et al., 2019). The increase in the active-escape bias is a direct consequence of the increased influence of the Pavlovian policy (***Figure 5f-i***, black line), which in turn is a consequence of weaker beliefs that either of the instrumental Go/No-Go actions will lead to the desired neutral outcome (cf. hopelessness) (***Figure 5f-i***, colored lines). The latter is a direct consequence of increased *k*, leading to an over-adjustment of beliefs after aversive outcomes. This also disrupts the agent’s ability to adapt to a changing environment because negative outcomes after the reversal become less surprising: this is reflected in reduced SAPEs for aversive outcomes and increased SAPEs for neutral outcomes (***Figure 5d***). Assuming SAPEs are computed in dPFC (Sales et al., 2019; Gläscher et al., 2010), this result would be consistent with empirical findings of increased dPFC response to wins vs. loses in suicide attempters (Olié et al., 2015) and reduced dlPFC activation in response to negative stimuli in suicidal ideation (Miller et al., 2018).

### 3.2 Multiple routes to an active-escape bias

While directly increasing learning from aversive outcomes (*k*) is one way to produce the effects associated with STB, there is a wider hypothesis space to be explored. To that end, we performed a more extensive investigation of the effects of other model parameters. In this context, it is important to note that the model exhibits a considerable degree of stochasticity when initiated with the chosen parameter configurations and thus, the results presented earlier in ***Figures 4*** and ***5*** are meant to be primarily illustrative. To reduce stochasticity and to obtain more robust behavioral results, now we used 400 trials with a reversal at 200 and ran 50 simulations for each parameter configuration. To visualize the results, we computed relevant task performance summary statistics (mean and standard error) for each parameter configuration (***Figure 6***). The first column in ***Figure 6*** simply reproduces the results in ***Figures 4*** and ***5***, showing that as we increase learning from negative outcomes, we reduce beliefs that instrumental actions will lead to the desired states (***Figure 6a***), which leads to an increase in the probability of the Pavlovian policy (***Figure 6e***), which in turn leads to a larger active-escape bias (***Figure 6i***). As a result of the increased biases, we also see a slight decrease in the overall performance accuracy (***Figure 6m***).

Reducing base belief decay (increasing parameter *m*) produces similar results of more negative beliefs, a higher probability of the Pavlovian policy and a stronger active-escape bias (***Figure 6b,f,j***). We also see a deterioration of the overall performance accuracy after the reversal, as the agent is slow to adapt to new contingencies (***Figure 6n***). Although very little research exists on reversal learning in suicidality, the latter result is in line with impaired reversal learning demonstrated in a reward/punishment probabilistic learning task in suicide attempters (Dombrovski et al., 2010).

A higher stress sensitivity (larger *c*) also produces the effects associated with STB: more negative beliefs (***Figure 6c***) lead to a higher probability of the Pavlovian policy (***Figure 6g***) and a stronger active-escape bias (***Figure 6k***). Finally, the overall performance accuracy (***Figure 6o***) shows a non-linear dependence on stress sensitivity, which is reminiscent of the inverted U-shaped relationship between stress and performance (Yerkes et al., 1908; Hebb, 1955). It is important to note that the *c* parameter features in the model twice: first, in the prior over outcomes, and second, in the learning rate after aversive outcomes. The decrease in the overall performance accuracy and the increase in the active-escape bias at very low values of *c* can be explained by the former role of this parameter. In other words, a small *c* means little motivation to prefer neutral outcomes (e.g., the aversive outcomes are not experienced as very aversive), which leads to a more random policy selection and thus effectively increases Pavlovian influences and reduces overall performance accuracy. In contrast, the increased active-escape bias associated with larger *c* values derives from parameter *c*‘s contribution to the learning rate. Interestingly, both reduced and increased distress tolerance have been associated with suicide risk: lower distress tolerance relates to psychological/social pain and contributes to suicidal ideation, while higher distress tolerance relates to physical pain and contributes to the acquired capability for suicide (see Liu et al. (2016) for a discussion). Our model simulations are agnostic to the nature of the aversive stimulus used and thus might be capturing both of these effects.

Reducing perceived controllability (increasing *w*_0_) is yet another way to produce the effects associated with STB. By way of a self-fulfilling prophecy, a reduced controllability threshold leads to more negative beliefs (***Figure 6d***), which induces increases in the Pavlovian policy probability (***Figure 6h***) and an active-escape bias (***Figure 6l***), as well as a slight decrease in the overall performance accuracy (***Figure 6p***).

### 3.3 Computational parameters reveal STB subtypes

**Figure 7:**
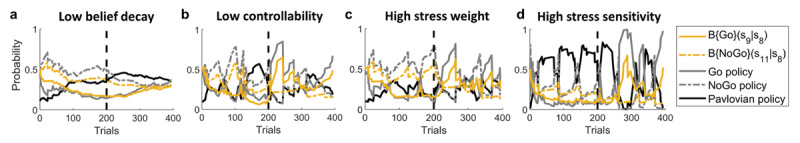
**Model simulations: trajectories of beliefs and policies under different parameter manipulations.** (**a**) low belief decay, *m =* 2, (**b**) low controllability, *w*_0_ = 0.8, (**c**) high stress weight, *k =* 1.1, (**d**) high stress sensitivity, *c =* 20. The other parameters were set to the same values as in Figure 6. All panels show trajectories of NGE/NE cue: where the cue is NGE before the reversal (the vertical dashed line) and GE after the reversal. Less variable rigid negative beliefs and Pavlovian policy in (a) could be associated with planful suicide attempts, whereas more variable beliefs and sudden increases in Pavlovian policy in (**b-d**) could be associated with more impulsive suicide attempts (Schmaal et al., 2020; Bernanke et al., 2017).

While all of the above parameter manipulations lead to similar mean behavioral effects, inspecting the time series reveals different dynamics of belief updating and policy probabilities (***Figure 7***). Using NGE/NE cue as an example, for high *m* values (low belief decay rate), we can see a very gradual progression towards more negative beliefs and an increased influence of the Pavlovian policy (***Figure 7a***). For high *w*_0_ (low controllability), high *k* (high stress weight) and high *c* (high stress sensitivity), we see increasingly larger and sudden spikes in Pavlovian biases. Considering the influence of the Pavlovian policy as a proxy for STB risk, the former scenario suggests a constantly increasing risk of STB and thus could be related to planful suicide attempts, while the latter scenario suggests an increased STB risk immediately after the occurrence of aversive events and could relate to impulsive suicide attempts. Bearing in mind our proposed links between the model parameters and the underlying neurocircuitry (***Figure 3***), these results are consistent with planful and impulsive suicide attempt subtypes, with the former being predominantly associated with dPFC activity and the latter being predominantly associated with vmPFC activity (Schmaal et al., 2020).

## 4 Discussion

In this paper, we presented a computational model of hopelessness and Pavlovian/active-escape bias in suicidality. With this model we showed that increased Pavlovian control and active-escape biases result from state hopelessness via the drive to maximize model evidence. Moreover, we proposed how hopelessness itself can arise from four mechanisms: (1) increased learning from aversive outcomes, (2) reduced belief decay in response to unexpected outcomes, (3) increased stress sensitivity and (4) reduced sense of stressor controllability. We also proposed how these alterations might relate to the neurocircuits implicated in suicidality. Specifically, we considered perturbations in the LC-NE system together with the Amy, the dPFC and the ACC, which mediate learning in response to acute stress and volatility, as well as perturbations in the DRN-5-HT system together with the vmPFC and the Amy, which regulate stress reactivity and its modulation by perceived controllability. We validated the model via simulations of an Avoid/Escape Go/No-Go task reproducing the active-escape biases reported by Millner and colleagues (Millner et al., 2019, 2018).

First, it is worthwhile to elaborate on what advantages and new insights our proposed model brings compared to previous modelling work. Millner et al. (2019) analyzed the increased active-escape bias in STB using a combined reinforcement learning – drift diffusion model (RL-DDM) and found that an increased active-escape bias can be explained by a bias parameter (aka a starting point in the DDM part of the model). This parameter was assumed to be constant throughout the task. In contrast, our proposed model offers a mechanistic explanation for how active-escape bias arises dynamically from learning about the state transition probabilities and balancing between instrumental and Pavlovian policies. Unlike in RL-DDM, in our model, Pavlovian and instrumental policies are represented explicitly. Importantly, this allowed us to relate state transition probabilities to state hopelessness (which is a central construct in suicidality research (Klonsky et al., 2018; May et al., 2020; Isometsä, 2014)), offering a possible operationalization of the hopelessness construct. Finally, using the active inference framework enabled us to propose several links (some more speculative than others) between the model variables and the underlying neurocircuitry, which could help bridge the explanatory gap between neurobiology and cognition in STB (see Limitations section for further discussion).

Our model simulation results offer a computational hypothesis space by identifying mechanistically distinct perturbations that lead to hopelessness and Pavlovian/active-escape biases associated with STB. These distinct pathways might also speak to different suicidality subtypes: impulsive versus planful (Schmaal et al., 2020; Bernanke et al., 2017). While all of the four parameter manipulations produced increased Pavlovian control and active-escape biases, examining the trajectories of belief updating revealed that reduced belief decay led to more gradual updates and more stable negative beliefs as well as more stable and elevated Pavlovian influences, which could be associated with more planful STB. The other three manipulations – reduced controllability of stressors, increased learning from aversive outcomes and increased stress sensitivity – resulted in increasingly variable belief updates with sudden spikes in Pavlovian biases after aversive outcomes, which could be associated with more impulsive STB. Considering the dPFC and the vmPFC as possible correlates of belief decay and controllability (and its effects on stress reactivity), respectively, our results are in agreement with neuroimaging studies associating disruptions in vmPFC activity with the impulsive STB subtype and the dPFC activity with the planful STB subtype (Schmaal et al., 2020).

While throughout the paper we have adopted a transdiagnostic view of STB, many mental disorders are known to increase suicide risk. Among all disorders, borderline personality disorder (BPD), depression, bipolar disorder, schizophrenia, and anorexia nervosa show the highest risk of suicide – between 10 to 45 times higher than the general population (Chesney et al., 2014). Comorbidities further increase suicide risk by inflicting higher levels of distress (Nock et al., 2010; Jylhä et al., 2016), with the majority of suicides being estimated to occur within a major depressive episode (Isometsä, 2014). Recent studies show preliminary evidence that suicide subtypes might cut across the current categories of disorders, with higher suicidal ideation variability (i.e. higher stress responsiveness) being associated with childhood physical abuse, aggression, and impulsivity in major depressive disorder (Oquendo et al., 2020) and with affective lability in BDP (Rizk et al., 2019). In a similar way, we might expect that the ways in which different mental disorders increase the risk of suicide could also map onto the different ways in which the effects associated with STB can emerge within our proposed model.

### 4.1 Model-based suicidality subtypes and personalized interventions

Being able to stratify the propensity for suicidal behavior into mechanistically distinct subgroups could help improve early interventions and treatment response prediction. Many different psychotherapies are applied in the context of suicidality, including the manualized therapies such as CBT, Dialectical Behavior Therapy (DTB), and mentalization-based therapy (MTB). However, evidence for the effectiveness of different psychotherapies is still scarce and it remains unclear which components of the therapies are most effective in reducing suicidality (Briggs et al., 2019; Ougrin et al., 2015; Weinberg et al., 2010). Moreover, the attempts to determine these unknowns are likely complicated by not accounting for the etiological heterogeneity in high suicide risk groups (Iyengar et al., 2018). Current neurobiological models of the mechanism of action of psychotherapy point to neural substrates of executive and semantic processes and highlight the vmPFC and its involvement in implicit emotion regulation as well as dPFC and its involvement in explicit behavioral control (Messina et al., 2016). This would map to the stressor controllability (vmPFC) and belief decay (dPFC) components in our proposed model and would suggest these parameters to be relevant when assessing, monitoring or optimizing the effectiveness of psychotherapy for a given suicidality subtype. For example, we could think of the controllability parameter as reflecting the level of felt control over one’s inner and outer life whereas the belief decay parameter could capture one’s ability to unlearn maladaptive beliefs through new experiences, behavior or cognitive reappraisal (Zilverstand et al., 2017).

When it comes to pharmacotherapy, sub-anesthetic doses of ketamine, a N-methyl-D-aspartate receptor (NMDAR) antagonist, is currently one of the most promising interventions for rapid reduction of STB, but only 55–60% of individuals respond with a complete remission (Wilkinson et al., 2018). The exact mechanism through which ketamine achieves its anti-suicidal and anti-depressant effects is still not fully understood (Riggs and Gould, 2021). Many hypotheses emphasize the importance of increased *α*-amino-3-hydroxy-5-methyl-4-isoxazolepropionic acid receptor (AMPAR) signalling, its involvement in bottom-up information transmission and a consequent increase in synaptic and spine plasticity (Zanos and Gould, 2018; Lengvenyte et al., 2019). Other recent in vivo microdialysis findings suggest ketamine-induced AMPAR signaling in LC and DRN as well as a subsequent release of NE and 5-HT in the mPFC to be necessary for the rapid antidepressant effects (López-Gil et al., 2019; Llamosas et al., 2019; Pham et al., 2017), also implicating prelimbic cortex (a homolog to Brodmann’s area 32 in the vmPFC) (PL)-DRN involvement in stressor controllability (Amat et al., 2016; Dolzani et al., 2018). A recent review also highlights the ACC to be playing a key role in mediating ketamine’s antidepressant effects (Alexander et al., 2021). The model we introduced here could help provide a more mechanistic understanding of how the changes in belief updating and possibly activity in these brain regions relate to reduced suicide risk.

Personalization of early interventions could also be improved by a more mechanistic understanding of sex differences as it relates to STB (Williams and Trainor, 2018). Females show a higher incidence of suicidal intent and suicide attempts, although the rate of completed suicides is much higher in males (2 to 5 times) (Freeman et al., 2017). Suicide risk factors have also been found to differ between the sexes (Oquendo et al., 2007). While multiple psychosocial factors are likely to be contributing to these differences (Canetto and Sakinofsky, 1998), sexual dimorphisms in the brain might play an important role as well (Pallayova et al., 2019). For example, structural and functional dimorphisms in the LC-NE system and its regulation by estrogen in females is associated with an increased susceptibility to hyperarousal (Bangasser et al., 2016), which itself has been linked to a higher risk of suicidal ideation (Steyn et al., 2013; Morabito et al., 2020; Dolsen et al., 2017). Preclinical studies also suggest important sex differences in how stressor controllability modulates stress reactivity. Unlike males, females do not seem to benefit from increased controllability, with the lack of engagement and structural plasticity within the PL-DRN pathway being a likely mechanism for these differences (Fallon et al., 2020). The model proposed here might help better understand how these differences impact stress reactivity and controllability, and how this affects response to ketamine as well as to other interventions (Fallon et al., 2020).

### 4.2 Limitations

While we have considered some of the most crucial neurocircuits and neuromodulatory systems at the overlap of stress response, aversive learning, behavioral control and STB, there remain other relevant regions to be considered (Schmaal et al., 2020; Lengvenyte et al., 2019). Of particular importance may be the lateral habenula (LHb), an epithalamic nucleus acting as a relay hub between forebrain and midbrain structures and playing a significant role in learning from non-rewarding and aversive experiences (Matsumoto and Hikosaka, 2009). The LHb is involved in stressor controllability effects via the DRN-5-HT system (Metzger et al., 2017) and is one of the locations targeted by ketamine that mediates the anti-depressant effects (Zanos and Gould, 2018; Yang et al., 2018a; Shepard et al., 2018). LHb activity has been associated with depressive symptoms of helplessness, anhedonia, and excessive negative focus (Yang et al., 2018b), while a recent study also reported higher resting state functional connectivity between LHb and several brain regions, including the amygdala, to be associated with STB independently of depressive symptoms (Ambrosi et al., 2019).

It is worth emphasizing that while a close consideration of the networks implicated in STB informed the construction of the model proposed here, the implementation of the model is not at the level of neural dynamics but rather at the level of higher-order computational mechanisms underwritten by such dynamics (cf. Marr’s levels of analysis (Marr and Poggio, 1976)). This means that the model variables might not necessarily neatly map onto distinct elements of the neurocircuitry but might interact with several other factors. For example, while we regard parameter *c* in the prior over outcomes to correspond to stress sensitivity and Amy activation, we could imagine other factors contributing to dispreference of the aversive outcome beyond its aversiveness per se, such as contextual factors relating to task engagement and a general motivation to do well in the task. Similarly, controllability threshold, *w*_0_, might reflect a combined influence of changes in vmPFC activation, its connectivity to the DRN, connectivity from the DRN to the Amy or even the LHb and the effects it exerts on the DRN-5-HT system. Future work, including modelling of the neural dynamics and gathering empirical data, will help clarify these relationships.

Related to the above limitations, it is also important to reiterate that the hypothesis space presented in this paper serves as a proof of concept and is not intended to be exhaustive. The emergence of STB risk factors in different contexts is most likely to involve other variables that we have not yet considered. Furthermore, our simulations explored only the simplest scenarios of varying one parameter at a time. Considering how these parameters interact provides another layer of complexity. For example, we could expect different subtypes of STB to be related not to a single parameter, but to a unique combination of multiple parameters, forming distinct clusters within the multidimensional parameter space. Future work with empirical data will allow for the further refinement of the hypothesis space and the delineation of different STB subtypes.

Furthermore, in the work presented here we have not explicitly addressed the distinction between suicide ideators and suicide attempters. Recent accounts of suicidality argue that suicidal ideation and the progression from ideation to attempts should be treated as separate processes (Van Orden et al., 2010; Klonsky and May, 2015; O’Connor and Kirtley, 2018; Bryan et al., 2020; Klonsky et al., 2018). The active inference framework might be well suited to study these distinctions as it explicitly models and factorizes inferences about the states of the world (cf. suicidal ideation) and action selection (cf. suicide attempt). This will be more thoroughly explored in future work.

Our proposed model and the insights it provides is also limited by the behavioral task to which the model was applied. In the task considered here, the stimulus is completely unambiguous and there is only one decision per trial to make. Notably, in the special case when outcomes unambiguously specify hidden states, active inference reduces to a simpler KL-control model (Friston et al., 2015), and is similar to model-based reinforcement learning models that are driven by reward maximization (see **Eq. (12)** in **S1 Appendix: full mathematical details of the model** for more detailed explanation). Introducing sensory uncertainty and multiple decisions would allow for the utilization of the unique aspects of active inference, namely epistemic action – a goal-directed sampling of information Friston et al. (2015). This would provide a more ecologically valid context to study the relationship between information sampling dynamics and STB. Such tasks would allow us to capture other phenomena relevant for STB, such as aversive generalization (how specific aversive events lead to negative beliefs about the world), its relationship to trauma, its effects on reduced problem-solving abilities (i.e. planning) and its influence on biases towards escape strategies (Linson and Friston, 2019; Linson et al., 2020).

Finally, it also important to point out that most of the model parameters that we focused on in this paper are not unique to the active inference framework. To explain the active-escape bias phenomenon, we had to introduce additional parameters and computations. Namely, we introduced the dependency of the learning rate on outcome values and the dependency of outcome values on controllability, with controllability being another addition in itself. Such a modelling approach where additional parameters and computations are added on top of the existing ones is not uncommon and has been applied in many reinforcement learning modelling approaches focused on Pavlovian and instrumental control mechanisms (e.g., Dorfman and Gershman, 2019; Guitart-Masip et al., 2012; Mkrtchian et al., 2017; Na et al., 2021; Grossman et al., 2021). However, it contrasts with most of the developments in active inference, where model extensions are derived from the free energy functional. While we justified the introduction of these computations by relying on a large body of literature investigating the mechanisms of interest, a more principled derivation of computations capturing these mechanisms might be possible.

## Data Accessibility Statements

General Matlab code implementing Active Inference can be found at *https://www.fil.ion.ucl.ac.uk/spm/software/spm12/*. Code from this toolbox (*spm_mdp_VB.m*) was modified to perform the simulations presented in this paper and can be found at: *https://github.com/frank-pk/STB_AEGNG_AI*.

## Additional File

The additional file for this article can be found as follows:

10.5334/cpsy.80.s1 S1 Appendix. Full mathematical details of the model.
