# Supplementary material for: A Computational Model of Hopelessness and Active-Escape Bias in Suicidality
Source: Comput Psychiatr. 2022 Mar 31;6(1):34–59. doi: 10.5334/cpsy.80 (PMC11104346; doi:10.5334/cpsy.80)
Supplement: S1 Appendix. — Full mathematical details of the model. [file cpsy-6-1-80-s1.pdf]

Appendix for "A neurocomputational model of suicidality: Aversive learning and active-escape bias"

This appendix provides full(er) mathematical details of the model presented in the main text. The implementation of the model is based on *spm_MDP_VB.m* - which is included in DEM toolbox of SPM12 (Wellcome Trust Centre for Neuroimaging, London, UK, http://www.fil.ion.ucl.ac.uk/spm). To keep the narrative clearer we will focus on the main components of the model highlighting the assumptions and additional computations that are unique to our implementation. Full derivations and explanations of active inference computations can be found in previous publications [1–3].

1 Variational Free energy

Perception and cognition is an inference process, where the hidden states of the world and one's internal model parameters, x, have to be inferred from sensory observations, o. This can be cast in Bayesian terms as:

$$P(x|o) = \frac{P(o|x)P(x)}{P(o)} \tag{1}$$

The exact posterior $P(x|o)$ is hard to compute, but can be approximated with some function $Q(x)$ by minimizing KL-divergence between $P(x|o)$ and $Q(x)$.

$$\begin{aligned} D_{KL}[Q(x)||P(x|o)] &= -\int dx\ Q(x)\ ln\left[\frac{P(x|o)}{Q(x)}\right] \\ &= -\int dx\ Q(x)\ \left[ln\left[\frac{P(x,o)}{Q(x)}\right] - lnP(x)\right] \\ &= -\int dx\ Q(x)\ ln\left[\frac{P(x,o)}{Q(x)}\right] + \int dx\ Q(x)\ lnP(o) \\ &= F + lnP(o) \quad . \end{aligned} \tag{2}$$

The first term variational free energy. The second term is log model evidence, which does not depend on $Q(x)$ and therefore remains constant. This means that by minimizing F, the agent can (1) minimize the difference between the unknown true posterior $P(x|o)$ and known approximation of that posterior $Q(x)$ and (2) simultaneously obtain a value of F which approximates model evidence. The expression of free energy can be further rearranged into a more convenient form:

$$\begin{aligned} F = -\int dx\ Q(x)\ ln[\frac{P(o,x)}{Q(x)}] &= E_{Q(x)}[lnQ(x) - lnP(o,x)] \\ &= \underbrace{D_{KL}[Q(x)||P(x)]}_{complexity} - \underbrace{E_{Q(x)}[lnP(o|x)]}_{accuracy} \quad , \end{aligned} \tag{3}$$

where the last equality gives a more intuitive expression of free energy (approximate model evidence) - balancing model accuracy and complexity.

2 Model factorization and parameterization

The agent's generative model can be formulated as Partially Observable Markov Decision Process (POMDP) (**Supplementary Fig. 1**).

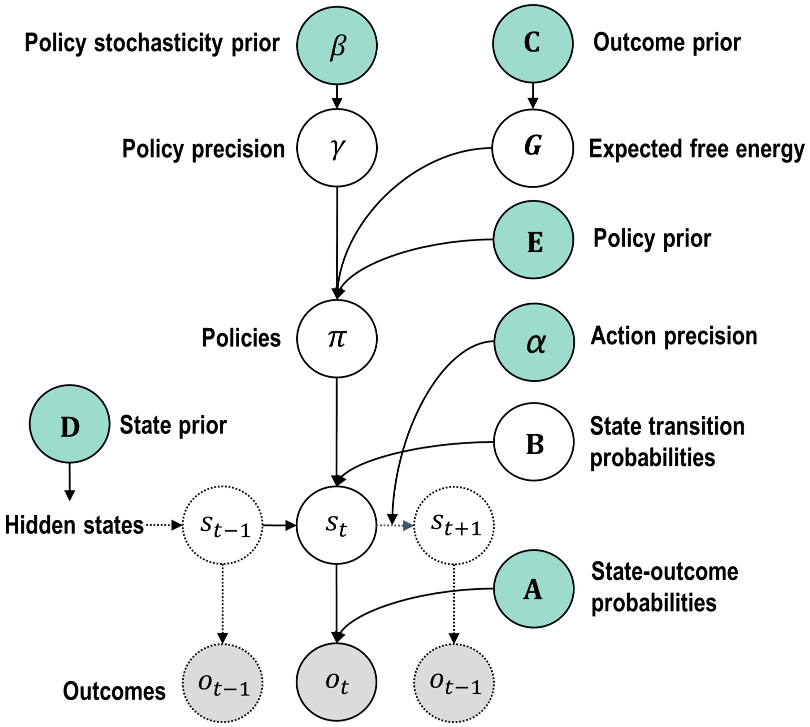

Supplementary Figure 1: **A graphical representation of the model.** Here we are showing a general formulation of the model with three points in time, the current one (t), the previous one ($t-1$) and the next one ($t+1$). Teal denotes fixed model parameters, white denotes random variables and model parameters that have to be inferred, while grey denotes sensory observations. Arrows indicate relationships between variables. Note that due to task constrains here we assume only learning in **B** to be relevant, while **A**, **C**, **D** and **E** are assumed to be fixed throughout the task.

Mathematically, the model can be expressed in terms of the joint probability over the sequence of observations $\tilde{o}$ and causes of those observations $x = (\tilde{s}, \pi, \theta)$, with $\tilde{s}$ being the sequence of states, π containing different policies (sequences of action) and $\theta = (a, b, c, d, e, \beta)$ containing a set of model parameters:

$$\begin{aligned} P(\tilde{o}, \tilde{s}, \pi, \theta) &= P(\pi)P(\theta)\prod_{t=1}^{T} P(o_t|s_t)P(s_t|s_{t-1}, \pi) \\ P(\tilde{o}, \tilde{s}, \pi, a, b, c, d, e) &= P(\pi)P(\boldsymbol{A})P(\boldsymbol{B})P(\boldsymbol{C})P(\boldsymbol{D})P(\boldsymbol{E})P(\gamma)\prod_{t=1}^{T} P(o_t|s_t)P(s_t|s_{t-1}, \pi) \end{aligned} \tag{4}$$

The agent's approximate posterior over hidden states and model parameters then takes the form of:

$$
\begin{aligned}
Q(\tilde{s}, \pi, \theta) &= Q(\pi)Q(\theta)\prod_{t=1}^{T} Q(s_t|\pi) \\
Q(\tilde{s}, \pi, a, b, c, d, e) &= Q(\pi)Q(\boldsymbol{A})Q(\boldsymbol{B})Q(\boldsymbol{C})Q(\boldsymbol{D})Q(\boldsymbol{E})Q(\gamma)\prod_{t=1}^{T} Q(s_t|\pi)
\end{aligned} \tag{5}
$$

$\boldsymbol{A}$ is a matrix of probabilities of different outcomes being observed at different states $P(o|s)$. Each column in $\boldsymbol{A}$ corresponds to a different state and is effectively a categorical distribution providing probabilities of different outcomes. The columns in $\boldsymbol{A}$ are Dirichlet distributions parameterized by concentration parameters, a, such that $P(\boldsymbol{A}) = Dir(a)$. The concentration parameters effectively reflect the number of times a particular combination of states and outcomes has co-occurred. All other matrices and vectors presented below are defined in the same way - as Dirichlet distributions parameterized by concentration parameters.

$\boldsymbol{B(u)}$ is a matrix of probabilities of state transitions $P(s_{t+1}|s_t, \pi)$ for action u (either Go or No-go, in our case) between time t and $t+1$. Each column is a categorical probability distribution for reaching different states given the current state. In addition to $\boldsymbol{B(u)}$, which corresponds to instrumental behavior, we also have an additional $\boldsymbol{B_0}$ matrix of transition probabilities that encode Pavlovian responses - active Go response when in the presence of an aversive stimulus and passive No-go response when the aversive stimulus is not present. Implementing Pavlovian control in this way is very similar to how habitual behavior has been implemented in previous publications [2].

$\boldsymbol{C}$ is a vector of log probabilities encoding the prior over outcomes $lnP(o)$, which effectively are preferred outcomes, because they guide policy selection, as will become clearer later. Here we assume all outcomes involving an aversive stimulus to be disprefered, having a value of $-c$ and the neutral outcomes having values of 0.

$\boldsymbol{D}$ is a vector of probabilities encoding the prior for the initial ($t = 1$) state $P(s_1)$. Here we set $\boldsymbol{D}$ to be uniform across the four possible initial hidden states. However, in the context of the task we are modelling, prior over the initial states has little influence because we assume no ambiguity in the observation likelihood $\boldsymbol{A}$ and thus cue information is fully sufficient for accurate state inference.

$\boldsymbol{E}$ is a vector of probabilities encoding the prior over policies $p(\pi)$. Here we have two instrumental policies (Go or No-go) and one Pavlovian policy and we set the prior to be uniform. β is a stochasticty prior that is used to inform precision of policy probabilities according to a gamma distribution $P(\gamma) = \Gamma(1, \beta)$. Throughout the simulations presented in this work, we keep β set to its default value of 1.

Now that we have specified our model, we can proceed to how (1) inference of states and (2) policy selection within trials as well as (3) model parameter updates across trials (i.e. learning) are all underwritten by free energy minimization.

3 Inference

3.1 States

Substituting the true and approximate posteriors in the free energy expression in Eq. (3) with the true posterior from Eq. (4) and the approximate posterior from Eq. (5) gives us:

$$
\begin{aligned}
F &= E_Q\left[ln\left(Q(\pi)Q(\theta)\prod_{t=1}^{T}Q(s_\tau|\pi)\right) - ln\left(P(\pi)P(\theta)\prod_{t=1}^{T}P(o_t|s_t)P(s_t|s_{t-1},\pi)\right)\right] \\
&= D_{KL}[Q(\pi)||P(\pi)] + D_{KL}[Q(\theta)||P(\theta)] + E_Q\left[\sum_{\tau=1}^{T} lnQ(s_\tau|\pi) - lnP(s_\tau|s_{\tau-1},\pi) - lnP(o_\tau|s_\tau)\right]
\end{aligned} \tag{6}
$$

The last term of Eq. (6) corresponds to free energy of hidden states:

$$
\begin{aligned}
F(\pi) &= \sum_\tau F(\pi,\tau) \\
&= \sum_\tau E_{Q(s_\tau|\pi)}\left[lnQ(s_\tau|\pi) - lnP(s_\tau|s_{\tau-1},\pi) - lnP(o_\tau|s_\tau)\right] \\
&= \sum_\tau \boldsymbol{s_\tau^\pi} \cdot \left[ln(\boldsymbol{s_\tau^\pi}) - ln(\boldsymbol{B_{\tau-1}^\pi})\boldsymbol{s_{\tau-1}^\pi} - ln(\boldsymbol{A}) \cdot o_\tau\right] \quad ,
\end{aligned} \tag{7}
$$

where the last equality is exactly the same as the one before, but in a shorthand notation, with $\boldsymbol{s_\tau^\pi} := Q(s_\tau|\pi)$ and with o_τ being a vector containing observed outcomes at time point τ. At the beginning of a trial when there is no previous state ($\tau = 1$), state transition matrix $\boldsymbol{B}$ is replaced by state prior $\boldsymbol{D}$:

$$
F(\pi,1) = \boldsymbol{s_1^\pi} \cdot \left[ln(\boldsymbol{s_1^\pi}) - ln(\boldsymbol{D}) - ln(\boldsymbol{A}) \cdot o_1\right] \tag{8}
$$

Differentiating $F(\pi)$ with respect to states and setting to zero gives an updated probability distribution over states:

$$
\boldsymbol{s_\tau^\pi} = \sigma\left(ln(\boldsymbol{B_{\tau-1}^\pi})\boldsymbol{s_{\tau-1}^\pi} - ln(\boldsymbol{A}) \cdot \boldsymbol{o_\tau} + ln(\boldsymbol{B_\tau^\pi})\boldsymbol{s_{\tau+1}^\pi}\right) \tag{9}
$$

At each time point, after new outcomes have been observed, $F(\pi)$ and $\boldsymbol{s_\tau^\pi}$ are computed iteratively until convergence.

3.2 Policies and action selection

After the expected states $\boldsymbol{s_\tau^\pi}$ are obtained, the next step is to determine relative probabilities that each of the policies (π) will lead to the desired outcomes ($\boldsymbol{C}$). This requires to first compute expected free energy $G(\pi)$ - free energy over future time points (from $t+1$ to time horizon T). Based on Eq. (3), we can write:

$$
\begin{aligned}
G(\pi) &= \sum_{\tau=t+1}^{T} G(\pi,\tau) \\
&= \sum_{\tau=t+1}^{T} E_{Q(o_\tau,s_\tau|\pi)}[lnQ(s_\tau|\pi) - lnP(o_\tau,s_\tau|\pi)]
\end{aligned} \tag{10}
$$

with some simplifying assumptions (see [2,4] for full derivation), the above can be re-expressed as:

$$
\begin{aligned}
G(\pi) &= \sum_{\tau=t+1}^{T} \underbrace{E_{Q(o_\tau|\pi)}[lnQ(o_\tau|\pi) - lnP(o_\tau)]}_{expected\ cost} + \underbrace{E_{Q(s_\tau|\pi)}[H[P(o_\tau|s_\tau)]]}_{expected\ ambiguity} \\
&= \sum_{\tau=t+1}^{T} \boldsymbol{o_\tau^\pi} \cdot [ln(\boldsymbol{o_\tau^\pi}) - \boldsymbol{C}] + \boldsymbol{s_\tau^\pi} \cdot \boldsymbol{H}
\end{aligned}
\tag{11}
$$

where the last equality is exactly the same as the one before, but in a shorthand notation. H in the first equality denotes entropy of the distribution $P(o_\tau|s_\tau)$ and $\boldsymbol{H}$ in the second expression is a vector consisting of the diagonal elements of $\boldsymbol{A}ln\boldsymbol{A}$, while $\boldsymbol{o_\tau^\pi} := \boldsymbol{A} \cdot \boldsymbol{s_\tau^\pi}$. The first term in the expression captures the divergence between preferred outcomes and outcomes predicted under each policy (i.e. expected cost or risk), while the second term captures the uncertainty about the future outcomes in relation to expected states (i.e. expected ambiguity). Because we assumed no ambiguity in the likelihood function $\mathbf{A}$, the expected ambiguity will always be equal across the policies. This means that differences in expected free energy across policies will be driven only by the expected cost term - with expected free energy being lower for policies which promise outcomes that match outcome priors.

To obtain policy updates, we first set the prior over policies to $P(\pi) = \sigma(ln\boldsymbol{E} - \gamma G(\pi))$ and use a shorthand for the posterior $\boldsymbol{\pi} := Q(\pi)$. Then, differentiating Eq. (6) with respect to $\boldsymbol{\pi}$ and setting to zero gives (see [2, 4] for full details):

$$
\boldsymbol{\pi} = \sigma(ln\boldsymbol{E} - \gamma\boldsymbol{G}(\pi) - \boldsymbol{F}(\pi)) \tag{12}
$$

Since policy precision γ itself has to be inferred, we differentiate free energy with respect to γ and set to zero. This gives:

$$
\begin{gathered}
\boldsymbol{\beta} = \beta + [\boldsymbol{\pi} - \boldsymbol{\pi_0}] \cdot \boldsymbol{G}(\pi) \\
\gamma = 1/\boldsymbol{\beta} \quad ,
\end{gathered}
\tag{13}
$$

where $\boldsymbol{\pi_0} := \sigma(ln\boldsymbol{E} - \gamma\boldsymbol{G}(\pi))$ and unbolded β is the stochasticity prior that is set as a model parameter ($\beta = 1$ throughout the simulations in this paper). Due to the interdependence, policy probability estimates and policy precision estimates are iterated recursively until convergence.

The obtained policy probabilities can then be integrated with expected states under each policy to obtain Bayesian Model Average (BMA) state expectations:

$$
\boldsymbol{S_\tau} = \sum_p \boldsymbol{\pi_\tau^p} \cdot \boldsymbol{s_\tau^p} \quad , \tag{14}
$$

where $\boldsymbol{\pi_\tau^p}$ denotes posterior policy probabilities and $\boldsymbol{s_\tau^p}$ denotes posterior state probabilities for policy p at time point τ.

The probabilities of different actions (u) are then obtained by assuming that the agent is more likely to select an action at time t that will bring about the outcome predicted by the BMA over states at time $t+1$:

$$
P(u|\boldsymbol{\pi}) \propto -D_{KL}(\boldsymbol{A}\boldsymbol{S_{t+1}}||\boldsymbol{A}\boldsymbol{B}(u)\boldsymbol{S_t}) \tag{15}
$$

Next, $P(u|\boldsymbol{\pi})$ is weighted by action precision:

$$
P(u|\alpha) = \sigma(\alpha \cdot P(u|\boldsymbol{\pi})) \quad , \tag{16}
$$

where α is action precision, with values less than 1 resulting in noisier actions and values more than 1 resulting in more precise actions (relative to the original $P(u|\boldsymbol{\pi})$). In the limit cases, very low

values would lead to random actions, whereas very high values would correspond to deterministic behavior always choosing action with the highest probability (as opposed to probability matching). Throughout the simulations in this paper α was set to 3.

Finally, the actions are chosen based on probability matching:

$$u = \begin{cases} Go, & if\ P(u = Go|\alpha) > n) \\ NoGo, & if\ P(u = Go|\alpha) < n) \end{cases} \tag{17}$$

Where $n \in [01]$ is a random number and is drawn for each trial from a uniform distribution.

4 Learning

At the end of each trial, after the actions are executed and the outcomes are observed, the agent updates the model parameters. Given the constrains of the task being modelled, here we choose to focus on how instrumental state transition matrices $\boldsymbol{B}(u)$ are learned and assume all other model parameters to be fixed. As described in the main text, updates are implemented via the Dirichlet concentration parameters:

$$b_i(u) = b_{i-1}(u) + \eta \sum_{\tau,p} \pi^p_{\tau-1}\ s^p_\tau \otimes s^p_{\tau-1} - \frac{b_{i-1}(u) - 1}{\lambda} \quad , \tag{18}$$

where i denotes the trial number, u denotes the action (Go or No-go), $\boldsymbol{s^p_\tau}$ is the posterior probabilities over all states for each policy p for time point τ. Note that in the current implementation, we only care about $\tau = 3$, because the transition between $t = 1$ and $t = 2$ does not depend on the agent's choices. $\boldsymbol{\pi}$ denotes posterior policy probabilities. (Note that here we use policy-blending to account for instrumental learning facilitated by Pavlovian responses [5]; in other words, the probability of Pavlovian Go or No-go response is combined with instrumental Go and No-go policy probabilities to obtain overall Go and No-go probabilities - stored in $\pi^p_{\tau-1}$ variable, when updating $\boldsymbol{b}s$).

The remaining two parameters η and λ control learning rate and decay rate, respectively. Following the work of Sales et al. [3], λ is assumed to depend on state-action prediction errors (SAPEs) and be associated with effective connectivity from the dPFC to the LC. The relationship between SAPE and λ is modelled using a logistic function:

$$\lambda = \lambda_{min} + \frac{\lambda_{max} - \lambda_{min}}{1 + e^{\ g(SAPE-m)}} \quad , \tag{19}$$

where g is the gradient, m is the midpoint, while λ_{min} and λ_{max} are minimum and maximum function values. Note that higher SAPE will result in smaller λ, which will result in more belief decay because λ is a denominator in the update equation **Eq. 18**. SAPE itself is defined as Kullback-Leibler (KL) divergence between BMA state distributions at successive time steps:

$$SAPE(t) = D_{KL}[(\boldsymbol{S^t_\tau})||\boldsymbol{S^{t-1}_\tau})] \quad . \tag{20}$$

In the simulations presented in this paper, SAPE is computed for t = 3, after the action (Go/No-go) is performed and only for predictions about the final states ($\tau = 3$).

Note, up until this point we have been following previous work in the implementation of the update equations. In what follows we introduce additional ad hoc computations for the purpose of understanding the effects seen in the Avoid/Escape Go/No-go task.

The first consideration comes from the fact that in addition to being sensitive to environmental change (i.e. volatility), the LC-NE system also coordinates aversive learning mediated by Amy-LC

connectivity [6, 7]. To capture these effects, we introduce a learning rate dependency on amygdala activity, which we associate with the preference against aversive outcomes encoded in the **C** vector:

$$\eta = 1 + k|\boldsymbol{C}(o)| \quad , \tag{21}$$

where $C(o)$ is the value of prior preference for outcome o - which is $-c$ for the aversive stimulus outcomes and 0 for the neutral outcomes. k is a scaling factor that could be associated with effective connectivity between the amygdala and the LC. Note that the learning rate dependence on valence that we introduce here is what enables the model to account for affective biases [8–11]. A more principled implementation of valence and its role in modulating the learning rate could depend on the rate of change of free energy over time [12].

Another addition to the model aims to account for how controllability of aversive outcomes inhibits amygdala activation via the serotonergic system involving vmPFC-DRN-Amy network [13, 14]. We implement this by modulating stress sensitivity c by a controllability parameter w:

$$c' = c^{(1-w)} \tag{22}$$

Note that in the limiting cases when there is no control ($w = 0$) c' is equal to c and when there is complete control ($w = 1$) c' is equal to 1. Controllability itself is assumed to depend on the mean of beliefs that the neutral outcome will be reached (i.e. based on state transition probabilities encoded in ACC) averaging across the 2 possible actions:

$$w_n = \frac{1}{|a|}\Sigma_a P(o_6 \cup o_8|s_n, a), \quad n \in [5, 6, 7, 8] \tag{23}$$

Where $|a|$ denotes the number of available actions, $P(o|s_n, a)$ is simply the product of the likelihood of observations **A** and the state transitions **B** and n denotes the four available states at $t = 2$, which correspond to the four different conditions. w_n. w_n effectively represents average probability of achieving the desired outcome associated with each condition and thus with each cue. Note that this is similar to the well established finding of vmPFC encoding expected value (see [15] for a review). Furthermore, such distinction between vmPFC, which encodes expected outcome (which we associate with controllability) and ACC, which encodes state transition probabilities (which we relate to hopelessness) is consistent with the finding that vmPFC encodes stimulus-based value and is more active during outcome phase (cf. stress response) and that ACC encodes action-based value and is active during both outcome and decision phases (cf. instrumental control and learning) [16]. The close relationship between subjective feeling of control and outcome valuation has also been demonstrated in recent studies [17, 18]. Relevantly, STB has been associated with reduced activation to expected value in vmPFC [19, 20].

Finally, to collectively account for any impairments for how w_n modulates stress response (i.e., any impairments along the vmPFC-DRN-Amy network), we transform w_n into the final estimate of controllability by entering it into a logistic function constrained by a controllability threshold w_0 (i.e. the midpoint of the logistic function) and a gradient g_w:

$$w = \frac{1}{1 + e^{\;-g_w(w_n - w_0)}} \quad . \tag{24}$$

Note that even though we have provided a reasonable theoretical justification for introducing the controllability component, it is a rather ad hoc addition to the otherwise computationally principled active inference framework. It is important to stress, however, that the simulation results that we present in the next section do not hinge on this additional computation, except for the results concerning controllability parameter itself.

References

[1] Karl Friston, Thomas FitzGerald, Francesco Rigoli, Philipp Schwartenbeck, and Giovanni Pezzulo. Active inference: a process theory. *Neural computation*, 29(1):1–49, 2017.

[2] Karl Friston, Thomas FitzGerald, Francesco Rigoli, Philipp Schwartenbeck, Giovanni Pezzulo, et al. Active inference and learning. *Neuroscience & Biobehavioral Reviews*, 68:862–879, 2016.

[3] Anna C Sales, Karl J Friston, Matthew W Jones, Anthony E Pickering, and Rosalyn J Moran. Locus coeruleus tracking of prediction errors optimises cognitive flexibility: An active inference model. *PLoS computational biology*, 15(1):e1006267, 2019.

[4] Lancelot Da Costa, Thomas Parr, Noor Sajid, Sebastijan Veselic, Victorita Neacsu, and Karl Friston. Active inference on discrete state-spaces: a synthesis. *Journal of Mathematical Psychology*, 99:102447, 2020.

[5] Peter Dayan, Yael Niv, Ben Seymour, and Nathaniel D Daw. The misbehavior of value and the discipline of the will. *Neural networks*, 19(8):1153–1160, 2006.

[6] Akira Uematsu, Bao Zhen Tan, Edgar A Ycu, Jessica Sulkes Cuevas, Jenny Koivumaa, Felix Junyent, Eric J Kremer, Ilana B Witten, Karl Deisseroth, and Joshua P Johansen. Modular organization of the brainstem noradrenaline system coordinates opposing learning states. *Nature neuroscience*, 20(11):1602, 2017.

[7] Heidi IL Jacobs, Nikos Priovoulos, Benedikt A Poser, Linda HG Pagen, Dimo Ivanov, Frans RJ Verhey, and Kâmil Uludağ. Dynamic behavior of the locus coeruleus during arousal-related memory processing in a multi-modal 7t fmri paradigm. *Elife*, 9:e52059, 2020.

[8] Erdem Pulcu and Michael Browning. Affective bias as a rational response to the statistics of rewards and punishments. *Elife*, 6:e27879, 2017.

[9] Erdem Pulcu and Michael Browning. The misestimation of uncertainty in affective disorders. *Trends in Cognitive Sciences*, 23(10):865–875, 2019.

[10] Tali Sharot and Neil Garrett. Forming beliefs: Why valence matters. *Trends in cognitive sciences*, 20(1):25–33, 2016.

[11] Neir Eshel and Jonathan P Roiser. Reward and punishment processing in depression. *Biological psychiatry*, 68(2):118–124, 2010.

[12] Mateus Joffily and Giorgio Coricelli. Emotional valence and the free-energy principle. *PLoS Comput Biol*, 9(6):e1003094, 2013.

[13] Steven F Maier and Martin EP Seligman. Learned helplessness at fifty: Insights from neuroscience. *Psychological review*, 123(4):349, 2016.

[14] Deborah Lucille Kerr, Donald George McLaren, Robin Michelle Mathy, and Jack B Nitschke. Controllability modulates the anticipatory response in the human ventromedial prefrontal cortex. *Frontiers in Psychology*, 3:557, 2012.

[15] Jaryd Hiser and Michael Koenigs. The multifaceted role of the ventromedial prefrontal cortex in emotion, decision making, social cognition, and psychopathology. *Biological psychiatry*, 83(8):638–647, 2018.

[16] Eliana Vassena, Ruth M Krebs, Massimo Silvetti, Wim Fias, and Tom Verguts. Dissociating contributions of acc and vmpfc in reward prediction, outcome, and choice. *Neuropsychologia*, 59:112–123, 2014.

[17] David S Stolz, Laura Müller-Pinzler, Sören Krach, and Frieder M Paulus. Internal control beliefs shape positive affect and associated neural dynamics during outcome valuation. *Nature communications*, 11(1):1–13, 2020.

[18] Kainan S Wang and Mauricio R Delgado. Corticostriatal circuits encode the subjective value of perceived control. *Cerebral Cortex*, 29(12):5049–5060, 2019.

[19] Vanessa M Brown, Jonathan Wilson, Michael N Hallquist, Katalin Szanto, and Alexandre Y Dombrovski. Ventromedial prefrontal value signals and functional connectivity during decision-making in suicidal behavior and impulsivity. *Neuropsychopharmacology*, 45(6):1034–1041, 2020.

[20] Alexandre Y Dombrovski and Michael N Hallquist. The decision neuroscience perspective on suicidal behavior: evidence and hypotheses. *Current opinion in psychiatry*, 30(1):7, 2017.
